# α5 Subunit-containing GABA_A_ receptors mediate a slowly decaying inhibitory synaptic current in CA1 pyramidal neurons following Schaffer collateral activation

**DOI:** 10.1016/j.neuropharm.2009.11.005

**Published:** 2010-03

**Authors:** Mariana Vargas-Caballero, Loren J. Martin, Michael W. Salter, Beverley A. Orser, Ole Paulsen

**Affiliations:** a Department of Physiology, Anatomy and Genetics, University of Oxford, Sherrington Building, Parks Road, Oxford OX1 3PT, United Kingdom; b Institute of Medical Science, University of Toronto, Medical Sciences Building University of Toronto, Toronto, M5S 1A8, Canada; c Department of Physiology, University of Toronto, Medical Sciences Building University of Toronto, Toronto, M5S 1A8, Canada; d Program in Neurosciences & Mental Health, Hospital for Sick Children, 555 University Avenue, Toronto, Ontario M5G 1X8, Canada; e University of Toronto Centre for the Study of Pain, University of Toronto, 124 Edward Street Room 374, Toronto, Ontario M5G 1G6, Canada; f Department of Anesthesia, University of Toronto, Canada, 150 College Street Toronto, Ontario M5S 3E2, Canada; g Department of Anesthesia, Sunnybrook Health Science Centre, 2075 Bayview Avenue, Toronto, Ontario M4N 3M5, Canada

**Keywords:** α5, GABA_A_ receptor, Hippocampus, Inhibition, Mouse, Rat, Schaffer collateral

## Abstract

GABA_A_ receptors that contain the α5 subunit (α5GABA_A_Rs) are highly expressed in the hippocampus, and have been implicated in learning and memory processes. They generate a tonic form of inhibition that regulates neuronal excitability. Recently it was shown that α5GABA_A_Rs also contribute to slow phasic inhibition of CA1 pyramidal neurons following local stimulation in the *stratum lacunosum moleculare*. However, it is unknown whether α5GABA_A_Rs can also be recruited indirectly by stimulation of Schaffer collaterals. Here, we studied GABAergic currents evoked by stimulation in the *stratum radiatum* of CA1 in the presence and absence of CNQX to block AMPA receptor-mediated excitation. We tested their sensitivity to gabazine and two drugs acting at the benzodiazepine site of α1/α2/α3 or α5GABA_A_Rs (400 nM zolpidem and 20 nM L-655,708, respectively). IPSCs evoked by stimulation in the *stratum radiatum* in the presence of CNQX were potentiated by zolpidem, blocked by 1 μM gabazine and were relatively insensitive to L-655,708 consistent with the lack of α5GABA_A_Rs. In contrast, IPSCs evoked by stimulation of Schaffer collaterals had a significant gabazine-insensitive component. This component was attenuated by L-655,708 and enhanced by burst stimulation. Furthermore, the L-655,708-sensitive current was absent in recordings from mice lacking α5GABA_A_Rs (*gabra*5^−/−^ mice). These results show that α5GABA_A_R-mediated phasic inhibition is activated by the Schaffer collateral pathway and provide evidence for activity pattern-dependent participation of α5GABA_A_Rs in inhibition.

## 1 Introduction

Synaptic inhibition in the hippocampus plays a crucial role in balancing and synchronising the activity of excitatory cells. γ-aminobutyric acid (GABA) released by inhibitory interneurons activates GABA_A_ receptors (GABA_A_Rs), and in most mature neurons, GABA causes a reduction of the postsynaptic cell excitability via hyperpolarising and/or shunting inhibition (for review, see Mann and Paulsen, 2007). GABA_A_Rs are Cl^−^ permeable, pentameric ionic channels that are formed from the combination of distinct subunits (α1–6, β1–3, γ1–3, δ, ɛ, θ, π, ρ1–3), and the majority of native combinations identified to date have a common 2:2:1 α/β/γ stoichiometry (reviewed in Wafford, 2005).

The targeting of pyramidal cells by inhibitory interneurons follows a highly organised pattern, and the vast majority of GABAergic interneurons target either the perisomatic or specific dendritic domains of pyramidal cells (Klausberger and Somogyi, 2008). However, the role of specific GABA_A_R subtypes expressed in distinct CA1 pyramidal cell compartments is still poorly understood. There is some evidence of a high correlation between presynaptic interneuron type and their specific GABA_A_Rs subunit targets (Nusser et al., 1996; Thomson et al., 2000). For example, in the neocortex, recordings from synaptically-connected pairs between GABAergic interneurons and pyramidal cells have demonstrated that dendritic targeting inhibitory neurons preferentially activate α5GABA_A_Rs, whereas those targeting the soma activate α1GABA_A_Rs (Ali and Thomson, 2008).

By identifying the GABA_A_R subtypes in different inhibitory pathways, it may be possible to pharmacologically target specific GABAergic networks in the hippocampus. Recent studies have started to dissect the contribution of GABA_A_Rs subtypes to different behaviours. Pharmacological tools in this area include the use of benzodiazepine derivatives or genetic modifications targeted at the benzodiazepine site, which is located at the interface of γ2 and α subunits (reviewed in Wafford, 2005). By altering the kinetics of single GABA_A_R channels, benzodiazepines enhance the effect of GABA and a behavioural readout can be obtained to interpret the function of targeted α subunits. For example, mice with a point mutation in the α1GABA_A_R subunit (His101Arg), which rendered it insensitive to diazepam, did not display the sedative and amnestic effects of benzodiazepines (McKernan et al., 2000; Rudolph et al., 1999). Conversely, inverse agonists acting at the benzodiazepine site (Tenen and Hirsch, 1980) inhibit the effect of GABA, and have effects opposite to those of the classical benzodiazepines. Using this approach, systemic application of the α3 inverse-agonist α3IA promoted anxiety-related behaviours in rodents (Atack et al., 2005).

α5GABA_A_Rs are of particular interest as they are highly expressed in the adult hippocampus both at synaptic and extrasynaptic sites (Sperk et al., 1997; Sur et al., 1999), in stark contrast to low expression levels in other brain areas. Consistent with their hippocampal localisation, behavioural studies using α5 subunit-specific inverse agonists and α5 subunit knock out mice strongly implicated α5GABA_A_Rs in the modulation of learning and memory (Collinson et al., 2002; Atack et al., 2006; Ballard et al., 2009). Therefore, α5GABA_A_Rs are currently considered as relevant targets for memory blocking drugs (Martin et al., 2009) and cognitive enhancing drugs with clinical applications such as in Alzheimer's disease patients, whose α5GABA_A_Rs are well preserved (Howell et al., 2000). However, the precise mechanisms underlying the regulation of hippocampal function by α5GABA_A_Rs are not known.

It is well established that extrasynaptic α5GABA_A_Rs can mediate a large component of tonic inhibition in the hippocampus (Caraiscos et al., 2004; Scimemi et al., 2005; Glykys and Mody, 2006; Prenosil et al., 2006). In contrast, the role of α5GABA_A_Rs in phasic inhibition remains poorly understood. Studies comparing spontaneous and locally-evoked inhibition between mice lacking α5GABA_A_Rs (*gabra*5^−/−^) and wild type (WT) mice suggested a negligible contribution of α5GABA_A_Rs to phasic inhibition (Collinson et al., 2002; Glykys and Mody, 2006). Other studies have suggested that α5GABA_A_Rs mediate a slowly decaying component of synaptic inhibition (GABA_A,slow_) (Prenosil et al., 2006; Zarnowska et al., 2009). Evoked GABA_A,slow_ potentials have only been observed following local extracellular stimulation at or near the *stratum lacunosum moleculare* (SLM) of the hippocampus (Pearce, 1993; Ouardouz and Lacaille, 1997; Zarnowska et al., 2009). Thus, GABA_A,slow_ has been proposed to modulate the activity of distal dendrites in hippocampal CA1 pyramidal neurons, and to mediate a component of synaptic inhibition activated by the direct input from the entorhinal cortex to the hippocampus at the SLM (Banks et al., 2000).

In order to understand the underlying mechanisms of α5GABA_A_Rs targeting cognitive enhancing drugs, it becomes important to establish whether in addition to their SLM activation, α5GABA_A_Rs are recruited by CA3 output via Schaffer collateral activity. Local stimulation at the *stratum radiatum* (SR) in CA1 has been reported to produce fast decaying IPSCs via perisomatic targeting inhibitory cells (Ouardouz and Lacaille, 1997) mediated by α1/α2/α3GABA_A_Rs (Thomson et al., 2000). However, under conditions of local stimulation, excitatory synaptic transmission is usually blocked with glutamate receptor antagonists. Feed-forward inhibition requires activation of afferent fibres to interneurons which in turn release GABA onto pyramidal cells (Alger and Nicoll, 1982). The Schaffer collaterals are likely to stimulate directly or indirectly a wide variety of interneurons that would not be reached by local stimulation. In the present study, we compared locally-evoked and Schaffer collateral-stimulated inhibitory currents. To determine whether α5GAB_A_Rs contribute to the evoked IPSCs we used the inverse-agonist L-655,708 in rats and *gabra*5^−/−^ mice. The results show that stimulation of Schaffer collaterals can activate a slowly decaying component of GABAergic inhibition, mediated by α5 subunit-containing GABA_A_ receptors, particularly following bursts of high-frequency stimulation of Schaffer collateral afferent input.

## 2 Methods

Animals were housed in groups with access to food and water *ad libitum*. The holding facilities maintained a temperature of approximately 22 °C, humidity of 60–70%, and a 12-h light/dark cycle. All animal care and experimental procedures were in accordance with the UK Home Office regulations under the Animals (Scientific Procedures) Act of 1986, and the Animal Care Committee of the University of Toronto.

### 2.1 Tissue preparation

Parasagittal slices containing the hippocampus were obtained from male Sprague Dawley rats (supplied by Harlan, Bicester, UK), or from *gabra*5^−/−^ mice (Collinson et al., 2002) and wild type (WT) littermates ranging from postnatal day 14 to 28. Rodents were anaesthetized with 5% isofluorane until breathing slowed down to approximately one breath per second, and stimulation of the limb withdrawal reflex no longer elicited a response. After decapitation, the brain was quickly removed into ice-cold artificial cerebrospinal fluid (aCSF), containing (in mM): NaCl, 126; KCl, 2.5; NaHCO_3_, 26; CaCl_2_, 2; MgCl_2_, 2; NaH_2_PO_4_, 1.25; glucose, 10, saturated with 95% O_2_/5% CO_2_, with a final pH of 7.2–7.4. Slices were prepared at 350 μm thickness using a Leica VT1000S microtome. Slices containing the hippocampal formation were trimmed from other brain regions and were maintained and recorded at room temperature (22–27 °C).

### 2.2 Electrophysiological recordings

After transferring a single slice to a submerged-style recording chamber, a monopolar stainless steel stimulation electrode (A-M Systems, Sequim, WA, USA) was placed into the SR of CA1 50–100 μm away from the *stratum pyramidale* (SP) for synaptic stimulation. Stimulation in the SR was carried out under two different conditions: firstly, to record GABA_A,local_, AMPA receptor-mediated excitation was blocked with CNQX while recording from a pyramidal cell. The stimulation electrode was placed approximately 100 μm lateral to the recorded cell to ensure stimulation of local interneurons. Secondly, to record inhibition elicited by the Schaffer collaterals (GABA_A,SC_) CNQX was not included. The stimulation electrode was placed approximately 300 μm lateral to the recorded cell to reduce local stimulation of GABAergic neurons in addition to afferent stimulation.

Experiments were performed in voltage clamp mode. The intracellular solution contained (in mM): Gluconic acid 70; CsCl 10; NaCl 5; BAPTA free acid 10; Hepes 10; QX-314 10; GTP 0.3; Mg-ATP 4; pH was titrated to 7.25 ± 0.05 with CsOH. The estimated final Cs concentration for the intracellular solution was ∼120 mM. The final osmolarity was 280 ± 5 mOsmol l^−1^. BAPTA was used to prevent Ca^2+^ dependent changes while measuring synaptic activity at depolarised membrane potentials. QX-314 blocks GABA_B_ receptor-mediated currents in addition to Na^+^ channels (Nathan et al., 1990). All voltage values were corrected for the liquid junction potential measured as 13 mV.

Whole-cell patch clamp recordings were obtained with 2–4 MΩ borosilicate pipettes from putative CA1 pyramidal cells identified by their location in the SP and by their shape.

The calculated E_Cl_ at room temperature was −56 mV, and AMPA receptor-mediated currents reversed near 0 mV. For this reason GABAergic currents were recorded at 0 mV, both for local and Schaffer collateral stimulation, so as to isolate them from AMPA receptor-mediated currents in the latter case. For recordings at voltages other than −70 mV, a voltage step from −70 mV to the test potential started 5 s before synaptic stimulation. Whole-cell recordings were made using an Axon Multiclamp 700B amplifier (Molecular Devices, Union City, CA, USA). Recordings were low-pass filtered at 2 kHz and digitised at 20 kHz with a National Instruments A/D board (Austin, TX, USA) using Ginj 1.0 software (courtesy of Hugh P. C. Robinson) for acquisition from within Matlab (Mathworks Ltd, Natick, MA, USA). Postsynaptic currents were evoked using a stimulus isolator unit (ISO-flex, A.M.P.I. Jerusalem, Israel) which delivered pulses of 100 μs duration in current mode; stimulation intensities ranged between 20 μA and 70 μA and the computer-controlled stimulation interval was 60 s. Drugs were applied after a stable baseline of 6–10 min (<10% drift allowed). Series resistance was not compensated during recordings. Series resistance was measured before each stimulation with a 5 mV, 50 ms step pulse. Recordings were terminated if series resistance (16 ± 6 MΩ) changed by more than 20%.

### 2.3 Data analysis

**Fig. 1:**
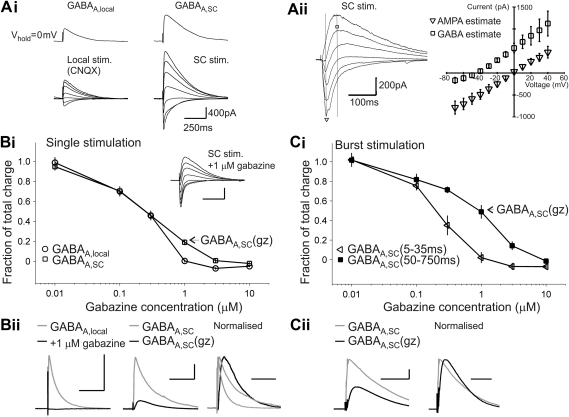
Locally-evoked and Schaffer collateral-stimulated GABA_A_R-mediated currents are differentially sensitive to gabazine. (Ai) Current traces showing voltage dependence of GABA_A_R currents evoked by synaptic stimulation in two different recording conditions. *Top traces*: inhibitory currents recorded at 0 mV; GABA_A,local_ in the presence of 10 μM CNQX and GABA_A,SC_. *Bottom traces*: Synaptic currents recorded at different holding potentials between −80 and +40 mV in steps of 20 mV. (Aii) Expanded traces for Schaffer collateral-stimulated currents (holding potentials of −70, −50, −30, −10, +10 and +40 mV) and current voltage relationship for estimated AMPAR (inverted triangles) and GABA_A_R (squares) mediated components (*n* = 5). Currents were measured at times indicated by vertical lines in example traces. (B, C) GABA_A_R currents in the presence of increasing concentrations of gabazine. (Bi) Concentration–response plot for GABA_A,local_ and GABA_A,SC_. Response is the charge transfer normalised to pre-gabazine control values. GABA_A,SC_(gz) indicates the 1 μM gabazine-resistant component of GABA_A,SC_. *Inset*: Example traces of Schaffer collateral-stimulated currents in 1 μM gabazine with holding potentials as in Ai. (Bii) Superimposed current traces, with and without 1 μM gabazine. Normalised traces for GABA_A,local_, GABA_A,SC_, and GABA_A,SC_(gz). (Ci) Concentration–response plot for the early and late components of GABA_A,SC_ elicited with burst stimulation (3 stimuli at 100 Hz). GABA_A,SC_(5–35 ms) and GABA_A,SC_(50–750 ms) were the measured charge transfers in the indicated time windows after stimulation. (Cii) Example traces of GABA_A,SC_(burst) recorded at 0 mV with and without gabazine. Normalised traces on the right. Scale bars in Bi-ii and Cii: 250 ms, 400 pA.

Data analysis was done using Matlab. Statistical testing was done using Matlab and SPSS software. Charge transfer was calculated by integrating the current responses from 5 to 750 ms following synaptic stimulation after leak subtraction (or 5–35 ms and 50–750 ms to separate early and late components in Fig. 1Ci). For comparison across experiments, synaptic peak current values or total charge transfer were normalised relative to the mean of baseline values obtained 5 min before drug application. For statistical comparison of drug effects, the average value of the last 4 min of recording was used. Data are presented as mean ± standard error of the mean (SEM) and are displayed in two-minute bin intervals. *N* values refer to the number of slices recorded. Example traces are the average of 3–5 traces (Gaussian-filtered at a corner frequency of 2 kHz). Statistical significance was assessed using Student's two-sample two-tailed *t*-test, one-way ANOVA or repeated measures (RM) ANOVA, with Bonferroni post-hoc corrections for multiple comparisons where appropriate. *P* < 0.05 was considered statistically significant.

### 2.4 Drugs

20 μM dl-2-amino-5-phosphonopentanoic acid was used to block *N*-methyl-d-aspartic acid (NMDA) receptors in all experiments. CNQX (6-cyano-7-nitroquinoxaline-2,3-dione disodium) was used to block AMPA receptor-mediated synaptic transmission for local stimulation of GABAergic interneurons. Gabazine (SR 95531 hydrobromide; 6-imino-3-(4-methoxyphenyl)-1(6*H*)-pyridazinebutanoic acid hydrobromide) is a competitive antagonist at GABA_A_Rs. L-655,708 (11,12,13,13a-tetrahydro-7-methoxy-9-oxo-9*H*-imidazo[1,5-a]pyrrolo[2,1-c][1,4]benzodiazepine-1-carboxylic acid, ethyl ester) is a partial inverse agonist at the benzodiazepine binding site of α5GABA_A_R; L-655,708 was initially dissolved to 10 mM in 1 N HCl, then diluted to 10 μM by adding H_2_O, and stored in frozen aliquots until used. Zolpidem (*N*,*N*,6-trimethyl-2-(4-methylphenyl)imidazol[1,2-a]pyridine-3-acetamide) is a benzodiazepine acting primarily at α1GABA_A_R, and with some affinity for α2/α3GABA_A_R at 400 nM. Zolpidem was dissolved at 100 mM in ethanol, frozen in aliquots, and diluted in aCSF just before use; equivalent amounts of ethanol were added in corresponding control experiments. All drugs described above were purchased from Tocris (Bristol, UK). Other chemicals were purchased from Sigma Aldrich (St. Louis, MO, USA).

## 3 Results

To measure synaptic GABA_A_R-mediated currents in CA1 pyramidal neurons in hippocampal slices, single cells were voltage clamped and brief extracellular stimuli delivered in the SR. First, to activate local GABAergic currents (referred to as GABA_A,local_), AMPA receptor-mediated excitation was blocked using 10 μM CNQX. GABA_A,local_ reversed close to E_Cl_ at −54 ± 3 mV (*n* = 5; data not shown; for example traces see Fig. 1Ai) and was seen as an outward current at 0 mV. At this holding potential, IPSCs had a time constant of decay (*τ*_decay_) of 163 ± 16 ms (*n* = 12).

Next, IPSCs were elicited by stimulating the Schaffer collaterals. For these recordings, CNQX was omitted from the extracellular solution. Biphasic responses comprising both glutamatergic and GABAergic currents were observed (Fig. 1Ai and Aii). At −70 mV, the slowly decaying inhibitory current was fully blocked by 10 μM gabazine leaving only a fast decaying AMPA current blocked by 10 μM CNQX). The excitatory component reversed near 0 mV as expected, and the inhibitory component reversed at −45 ± 1 mV (*n* = 5; Fig. 1Aii). The isolated GABA_A_R-mediated component was recorded at a holding potential of 0 mV (referred to as GABA_A,SC_, Fig. 1Ai). The decay time was significantly longer than that for GABA_A,local_ (GABA_A,SC_, *τ*_decay_ = 277 ± 29 ms, *n* = 10; *t*-test, *P* < 0.001). The ratio of current observed at 500 ms over 15 ms after stimulation (*I*_500ms_/*I*_15ms_) showed that GABA_A,local_ current had decayed to approximately 5% of its peak value after 500 ms, while a substantial fraction of GABA_A,SC_ could still be observed (GABA_A,local_, *I*_500ms_/*I*_15ms_ = 0.05 ± 0.01, *n* = 11; GABA_A,SC_, *I*_500ms_/*I*_15ms_ = 0.32 ± 0.06, *n* = 6; *t*-test, *P* < 0.001).

Both GABA_A,local_ and GABA_A,SC_ decayed significantly faster at −70 mV than at 0 mV (*τ*_decay_ = 70 ± 5 ms, *n* = 11, and 180 ± 10 ms, *n* = 6, respectively, *t*-test for both, *P* < 0.01). The slower decay of GABA_A,local_ and GABA_A,SC_ at 0 mV is consistent with previous reports showing that GABA_A_R currents decay more slowly at depolarised potentials as receptors unbind agonist at a slower rate (Mellor and Randall, 1998; Burgard et al., 1999). The currents observed at −70 mV were slower than those reported in some previous studies. Two factors might have contributed to the slower kinetics observed in our experiments: the use of BAPTA and the use of Cs-gluconate. Slower IPSC decay has been observed both during recording with BAPTA (Banks and Pearce, 2000) and with Cs-gluconate (Stepanyuk et al., 2002).

α5GABA_A_Rs are widely expressed in the SR subfield of the hippocampal CA1 area (Sperk et al., 1997; Sur et al., 1999), however their synaptic contribution has not been observed while stimulating locally in the SR. We therefore asked whether α5GABA_A_Rs can contribute to GABA_A,SC_. As a first pharmacological approach we tested the gabazine sensitivity of GABA_A_R currents, as it has been previously reported that a tonic inhibitory conductance mediated by α5GABA_A_Rs is resistant to 1 μM gabazine (Bai et al., 2001; Caraiscos et al., 2004).

To compare the gabazine sensitivity of locally-evoked and Schaffer collateral-stimulated currents, IPSCs were recorded in the presence of increasing concentrations of gabazine (Fig. 1Bi). GABA_A,local_ currents were abolished by 1 μM gabazine (initial amplitude, 863 ± 144 pA, *n* = 11) while a significant fraction of GABA_A,SC_ remained under these conditions (19 ± 3% of GABA_A,SC_ of control charge transfer remaining in 1 μM gabazine; *n* = 6, initial amplitude, 1261 ± 57 pA). This component is referred to as GABA_A,SC_(gz) Example traces are shown in Fig. 1Bii.

The time from stimulation to peak (Table 1) was significantly different for GABA_A,local_, GABA_A,SC_, and GABA_A,SC_(gz) (one-way ANOVA; *F*_2,21_ = 128; *P* < 0.001; post-hoc *P* < 0.01 for all comparisons). Both GABA_A,SC_ and GABA_A,SC_(gz) showed slower kinetics compared to GABA_A,local_ (Table 1, 10–90% rise time; one-way ANOVA; *F*_2,21_ = 51; *P* < 0.001; post-hoc *P* < 0.05 for all comparisons). The longer time-to-peak seen for GABA_A,SC_(gz) was additionally due to longer latency (Table 1, time from stimulus to 10% amplitude; one-way ANOVA; *F*_2,21_ = 75; *P* < 0.001; post-hoc comparisons, GABA_A,SC_(gz) *versus* GABA_A,local_ and GABA_A,SC_(gz) *versus* GABA_A,SC_, both *P* < 0.001; GABA_A,local_ *versus* GABA_A,SC_, *P* = 0.22).

As bursting input from the Schaffer collateral could be necessary for the firing of dendritic targeting interneurons (Maccaferri and Dingledine, 2002), we next asked whether bursting activity in this pathway could enhance GABA_A,SC_(gz). Indeed this component became prominent with burst stimulation (3–4 stimuli at 100 Hz) as 49 ± 7% of inhibitory charge transfer remained in 1 μM gabazine (Fig. 1Ci). Remarkably, the early component (5–35 ms after stimulation) of burst responses was almost as sensitive to 1 μM gabazine as GABA_A,local_ (fraction remaining in 1 μM gabazine: GABA_A,local_, 0.4 ± 1%, *n* = 6; GABA_A,SC_ (burst, 5–35 ms), 1 ± 5%, *n* = 6; *t*-test, *P* = 0.73; example traces in Fig. 1Cii). A significant effect of gabazine inhibition was observed for all recording conditions (GABA_A,local_, GABA_A,SC_ and GABA_A,SC(burst)_; RM ANOVA. Charge transfer as the between-subjects factor, and dose as within-subject factor; *F*_2,19_ = 25; *P* < 0.001; post-hoc comparisons showed that at 1 μM gabazine, each group was different from the others, *P* < 0.001).

**Fig. 2:**
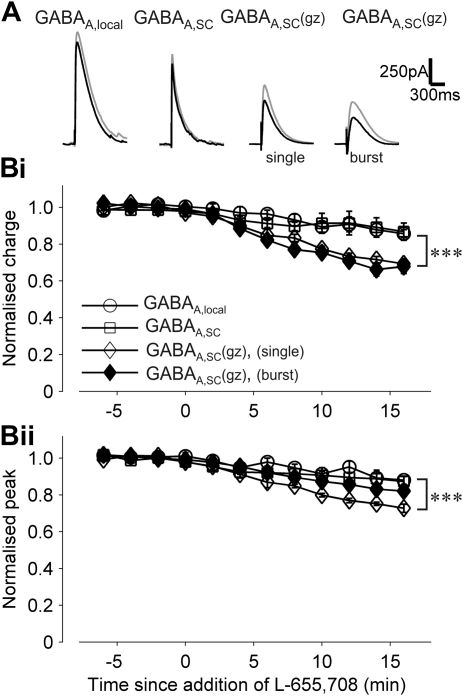
The α5 inverse-agonist L-655,708 inhibits GABA_A,SC_(gz). (A) Superimposed example traces of GABA_A_R currents before (grey) and after (black) application of L-655,708. (B) Normalised charge transfer (i) and peak current (ii) measured during baseline and after bath application of L-655,708 at time = 0. ***P < 0.001.

To test whether α5GABA_A_Rs contribute to the inhibitory currents described above we next investigated the effect of L-655,708, which is an inverse-agonist selective for α5GABA_A_R subunits in rats, mice and humans at concentrations below 20 nM (Atack et al., 2006). After a stable baseline recording, 20 nM L-655,708 was added to the extracellular solution. The effects of L-655,708 on charge transfer and peak amplitude were estimated for GABA_A,local_, GABA_A,SC_ and GABA_A,SC_(gz), with either single or burst stimulation for GABA_A,SC_(gz) (Fig. 2A). In all conditions, the average charge transfer after addition of L-655,708 was significantly different from baseline, but stronger effects were observed for GABA_A,SC_(gz) for both single and burst stimulation (percentage of reduction: GABA_A,local_, 13 ± 5%, *n* = 7; GABA_A,SC_, 12 ± 5%, *n* = 9; GABA_A,SC_(gz) (single), 30 ± 2%, *n* = 6; GABA_A,SC_(gz) (burst), 33 ± 3%, *n* = 7; Fig. 2B). A significant effect on peak was also observed for GABA_A,SC_(gz) (single) (*t*-test, *P* < 0.01), but did not reach significance for GABA_A,SC_(gz) (burst) (*t*-test, *P* = 0.07).

**Fig. 3:**
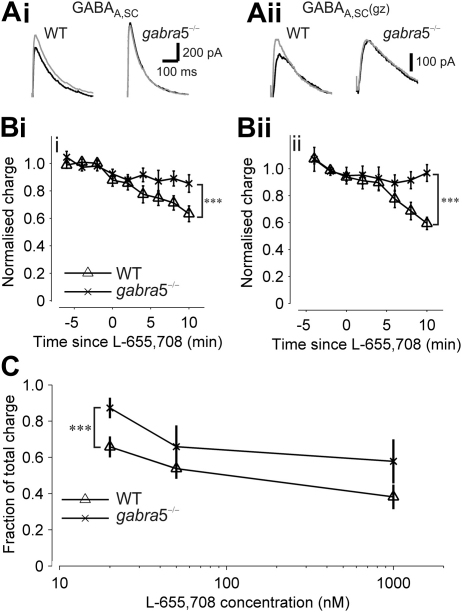
L-655,708 (20 nM) is specific for the GABA_A_ receptor α5 subunit. (A) Superimposed example traces of GABA_A,SC_ (i) and GABA_A,SC_(gz) (ii) before and after application of 20 nM L-655,708 in wild type and *gabra*5^−/−^ mice. (B) Normalised values for GABA_A_R charge transfer measured during baseline and following application of L-655,708 at time 0 for GABA_A,SC_ (i) and GABA_A,SC_(gz) (ii). (C) Concentration-response plot of GABA_A,SC_ for 20, 50 and 1000 nM L-655,708 in wild type and *gabra*5^−/−^ mice. ***P < 0.001.

To corroborate our findings on the effects of L-655,708 on GABA_A_R-mediated currents, and to test the specificity of L-655,708 for α5GABA_A_Rs, we next used *gabra*5^−/−^ mice and WT littermate control mice (Collinson et al., 2002). In this set of experiments, GABA_A,SC_ currents from WT mice showed a significant reduction from baseline following application of 20 nM L-655,708 (GABA_A,SC_, 33 ± 4% reduction, *n* = 5; GABA_A,SC_(gz), 36 ± 4% reduction, *n* = 7; *t*-test, *P* < 0.001 for both), whereas L-655,708 did not significantly alter the charge transfer for GABA_A,SC_ and GABA_A,SC_(gz) currents in *gabra*5^−/−^ slices (Fig. 3A, B; GABA_A,SC_, *n* = 7; *t*-test, *P* = 0.11; GABA_A,SC_(gz), *n* = 7; *t*-test, *P* = 0.20).

**Fig. 4:**
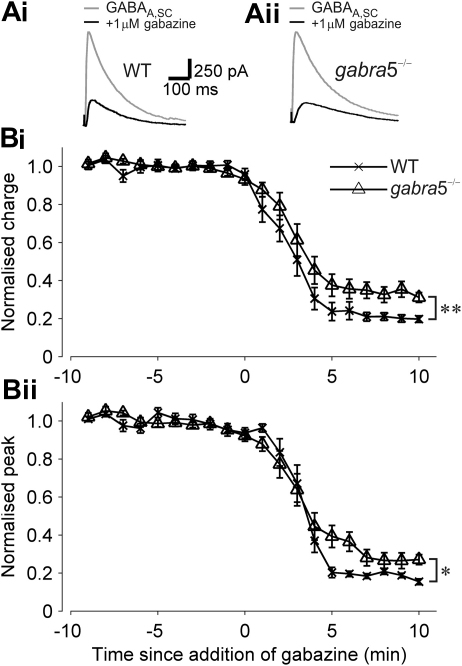
GABA_A_ receptor-mediated currents in *gabra*5^−/−^ mice and WT mice are differentially sensitive to 1 μM gabazine. (A) Superimposed example traces of GABA_A,SC_ currents before (grey) and after (black) application of 1 μM gabazine. Recordings from WT (i), and *gabra*5^−/−^ mice (ii). (B) Normalised values for charge transfer (i) and peak current (ii) measured during baseline and following application of 1 μM gabazine at time 0. *P < 0.05; **P < 0.01.

We additionally tested whether L-655,708 could be used as a selective inhibitor of α5GABA_A_Rs at concentrations higher than 20 nM during our recording conditions. We found that L-655,708 at 1 μM concentration, and even at concentrations as low as 50 nM, inhibited the GABA_A_R current in WT mice to a similar extent to that seen in *gabra*5^−/−^ mice (no significant difference WT *versus gabra*5^−/−^, *n* = 7 and *n* = 5, respectively: 50 nM, *P* = 0.33, and 1 mM, *P* = 0.16; Fig. 3C), suggesting that L-655,708 is not selective for α5GABA_A_Rs at concentrations above 20 nM, and thus limiting the selective inhibition of the α5GABA_A_R-mediated current to no more than ∼30%. Further analysis to compare GABA_A,SC_ currents recorded from WT and *gabra*5^−/−^ mice revealed that the fraction of GABA_A,SC_ charge transfer that persisted after the application of 1 μM gabazine was larger in WT mice than in *gabra*5^−/−^ mice (34.4 ± 4%, *n* = 11 *versus* 20.6 ± 2%, *n* = 10, respectively, *t*-test, *P* < 0.01; Fig. 4Ai, Aii, Bi). Also, the peak current was significantly different between *gabra*5^−/−^ and WT mice (*t*-test, *P* < 0.05; Fig. 4Ai, Aii, Bii).

**Fig. 5:**
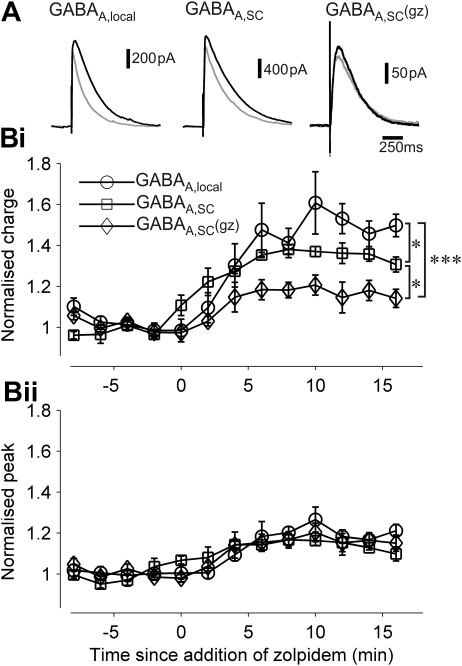
The enhancing effect of zolpidem is larger on GABA_A,local_ and GABA_A,SC_ than on GABA_A,SC_(gz). (A) Superimposed example traces of GABA_A_R currents before and after application of zolpidem (400 nM). From left to right: GABA_A,local,_ GABA_A,SC_, and GABA_A,SC_(gz) with corresponding symbols in B. (B) Normalised values of charge transfer (i) and peak current (ii) measured during baseline and following application of zolpidem at time 0. Significant difference of charge transfers but not peak currents between baseline and the last 4 min of recording for the three recording conditions. *P < 0.05; ***P < 0.001.

Finally, to test whether the GABA_A_R currents described above are mediated in part by α1/α2/α3GABA_A_Rs receptors we tested the effects of 400 nM zolpidem (Fig. 5). As expected, zolpidem increased the charge transfer of GABA_A,local_ currents (48 ± 6%, *n* = 7). Also, both GABA_A,SC_ and GABA_A,SC_(gz) charge transfer was enhanced (GABA_A,SC_ by 33.3 ± 4%, *n* = 5, and GABA_A,SC_(gz) by 16 ± 5%, *n* = 5; Fig. 5A, B). Zolpidem had a significantly different effect on currents under all recording conditions (GABA_A,local_, GABA_A,SC_ and GABA_A,SC_(gz); one-way ANOVA, *F*_2,32_ = 21.6, *P* < 0.001; post-hoc comparisons with significant differences as shown in Fig. 5Bi).

A slight increase in peak amplitude from baseline values was observed in all three recording conditions with no significant difference among them (Fig. 5Bii). The large increase in charge transfer and small change in peak amplitude in response to zolpidem have been previously observed for miniature postsynaptic potentials (Perrais and Ropert, 1999). In total, our results suggest that α5GABA_A_Rs contribute significantly to GABA_A,SC_.

## 4 Discussion

Previous work investigating the subunit composition of synaptic GABA_A_ receptors has measured spontaneous or stimulus-evoked GABAergic inhibition in the hippocampus while blocking glutamatergic excitation. In the present study, we describe the distinct pharmacology of locally-evoked *versus* Schaffer collateral-stimulated GABA_A_R currents. GABA_A,local_ currents were eliminated by 1 μM gabazine and were markedly enhanced by 400 nM zolpidem, whereas GABA_A,SC_ currents were less sensitive to 1 μM gabazine and zolpidem. The gabazine-insensitive component of GABA_A,SC_ showed the greatest reduction by L-655,708 and was relatively insensitive to zolpidem, suggesting a significant proportion of α5GABA_A_R. Furthermore, the gabazine-insensitive current was markedly enhanced following burst stimulation of Schaffer collaterals. Finally, we confirmed the specificity of L-655,708 on GABA_A,SC_ and the gabazine-insensitive currents by recording from WT and *gabra*5^−/−^ mice.

IPSCs recorded in CA1 pyramidal neurons evoked by local stimulation at the SR were likely generated by perisomatic targeting interneurons, including basket cells (Ouardouz and Lacaille, 1997). This GABA_A,local_ current had a *τ*_decay_ of 163 ms at 0 mV. In experiments designed to study Schaffer collateral-stimulated currents, the IPSCs decayed with a strikingly slow time constant (277 ms). GABA_A,local_ and GABA_A,SC_ also showed significantly different time-to-peak, and 10–90% rise time. Previous studies have already shown evidence for such fast and slow components of synaptic inhibition (Pearce, 1993; Banks et al., 1998). Whereas there was no significant difference in latency between GABA_A,local_ and GABA_A,SC_, suggesting that stimulation at the SR could also trigger direct activation of interneurons, GABA_A,SC_(gz) also showed a longer latency, suggesting that under these conditions, a delay in activation of presynaptic interneurons contributed to the longer time-to-peak.

Several possible explanations could account for the late peak and surprisingly slow decay of GABA_A,SC_ compared to GABA_A,local_. Firstly, using fast perfusion on excised patches from cells expressing recombinant receptors, it has been shown that subunit composition can affect decay time (Tia et al., 1996; Burgard et al., 1999). Secondly, the time course of GABA concentration at the release sites would affect the response kinetics, for example the rise time responses for extrasynaptic receptors could be slowed down as has been suggested for glutamatergic synapses (Scimemi et al., 2004). Thirdly, long exposure to GABA, either in the synaptic cleft or in extrasynaptic space, could produce the reactivation of synaptic receptors as previously shown by modifying GABA uptake kinetics (Roepstorff and Lambert, 1994). Recent studies using somatic recordings in anatomically-identified connected cell pairs suggest that the time course of inhibitory responses can indeed be determined both by subunit composition and by distinct transmitter release transients (Szabadics et al., 2007; Ali and Thomson, 2008), but we cannot exclude the possibility that dendritic filtering contributes to the slow kinetics as recorded at the soma. A further possibility with potential physiological significance is that the slow rise and decay kinetics we observed for GABA_A,SC_ is due to the pattern of activation of GABAergic interneurons. For example, late-firing dendritic targeting interneurons have been shown to generate slow GABAergic events in CA1 pyramidal neurons (Maccaferri and Dingledine, 2002). Furthermore, bursts of GABA release produced by several action potentials or slow asynchronous release (Hefft and Jonas, 2005) could contribute to slow kinetics.

GABA_A,SC_ and GABA_A,local_ showed different sensitivities to gabazine. We consistently found that approximately 20% of GABA_A,SC_ charge transfer evoked by single stimulation was insensitive to 1 μM gabazine, and this fraction increased to ∼50% when using burst stimulation. We interpreted this finding as an indication of a component mediated by α5GABA_A_Rs as it has been previously shown that the tonic current mediated by these is not sensitive to 1 μM gabazine (Bai et al., 2001; Caraiscos et al., 2004). We obtained further evidence that α5GABA_A_Rs are activated by Schaffer collateral stimulation by studying the effects of L-655,708 on the isolated GABA_A_R components described above. GABA_A,SC_(gz) produced either by single or burst stimulation was reduced by 30% and there was no significant difference in the amount of reduction using either type of stimulation (*P* = 0.27) as would be expected if in both cases the synaptically evoked current is mediated by a similar proportion of α5GABA_A_Rs. L-655,708 also produced a significant but smaller effect on GABA_A,SC_ and GABA_A,local_. In previous studies, spontaneous GABA_A,local_ currents were found not to contain an α5GABA_A_R-mediated component (Caraiscos et al., 2004; Glykys and Mody, 2006; Zarnowska et al., 2009), however, it is likely that the sorting of populations of spontaneous IPSCs by their decay time for analysis could limit the detection of a slow component in GABA_A,local_ currents. Furthermore, it is likely that the extracellular stimulation used to produce GABA_A,local_ under our experimental conditions would recruit interneurons other than perisomatic targeting interneurons. It is also possible that gabazine, as a competitive antagonist, could be displaced from GABA_A_ receptors by released GABA under our recording conditions for GABA_A,SC_(gz). Although we do not have evidence to discard this possibility, the effects of 20 nM L-655,708 on GABA_A,SC_ in both rat and mouse recordings without the use of gabazine strongly support a specific α5GABA_A_R-mediated component.

We corroborated the specificity of 20 nM L-655,708 on GABA_A,SC_ and GABA_A,SC_(gz) currents using *gabra*5^−/−^ mice and WT littermate controls. There was a one third reduction in the GABA_A,SC_ and GABA_A,SC_(gz) currents from initial control values following application of L-655,708 in WT mice, and no significant reduction in *gabra*5^−/−^ mice. *In vitro* analysis of L-655,708 activity on recombinant human GABA_A_Rs showed a maximum inhibition of 20% of the α5GABA_A_R current (Atack et al., 2006). Consistent with this we found in both rat and mouse recordings that 20 nM L-655,708 inhibited approximately 30% of GABA_A,SC_(gz).

As a final test to probe the composition of GABA_A_Rs mediating the early and late components of inhibition, we used 400 nM zolpidem which is a highly potent and selective benzodiazepine, with α5GABA_A_R sparing properties (α5GABA_A_R, *K*i > 15 μM, reviewed in Sieghart, 1995). GABA_A,local_ was markedly enhanced after application of zolpidem with a three fold increase in charge transfer compared to GABA_A,SC_(gz). In comparison, GABA_A,SC_ showed only a two fold increase compared to GABA_A,SC_(gz). We interpret this result to suggest that local stimulation in SR near SP in the absence of fast synaptic excitation mostly activates perisomatic targeting interneurons, which selectively activate α1, α2 and α3GABA_A_Rs (Thomson et al., 2000), and that activation of Schaffer collaterals not only stimulates perisomatic targeting cells but also dendritic targeting interneurons, some of which specifically target α5GABA_A_Rs. Therefore, the combined results using 400 nM zolpidem and 20 nM L-655,708 suggest that GABA_A,SC_ contains a significant population of α5GABA_A_Rs. Thus, the present observations highlight the strong activation of α5GABA_A_Rs following Schaffer collateral stimulation in the hippocampus and the pharmacological analysis of GABA_A,SC_(gz) suggests a particularly robust activation during bursting activity.

Our results are consistent with previous suggestions that slow inhibition is mediated by a specific subpopulation of interneurons and molecularly distinct receptors (Banks et al., 1998; Pearce, 1993; Zarnowska et al., 2009). The widespread localisation of α5GABA_A_Rs at dendritic sites (Sperk et al., 1997) suggests that they are involved in gating dendritic excitability, for example they could be involved in regulating the generation of dendritic spikes such as those observed *in vivo* during sharp waves (Kamondi et al., 1998). Furthermore, the time course similarity of GABA_A,SC_ and GABA_A,SC_(gz) to the decay kinetics of NMDA receptors (Vicini et al., 1998) makes them well suited for inhibition of the induction of long-term potentiation by providing a shunting inhibitory effect on the NMDAR current (Staley and Mody, 1992).

The higher acquisition rate in associative memory tasks observed in mice after systemic application of the inverse-agonist L,655,708 or in *gabra*5^−/−^ mice could be the expression of plasticity produced by enhanced dendritic excitation. In order to understand the cellular and network mechanisms that lead to enhanced learning after reduction of α5GABA_A_Rs function it will be necessary to study the requirement for activation/silencing of inputs targeting α5GABA_A_Rs on hippocampal pyramidal neurons by afferent inputs to the hippocampus and their timing in relation to the timing of somatic and dendritic spikes. Here we provide evidence of activity pattern-dependent feed-forward activation of α5GABA_A_Rs in CA1. Testing the role of α5GABA_A_Rs in controlling the generation of dendritic spikes or in the fine tuning of long-term potentiation awaits the broader availability of more potent and selective drugs acting on α5GABA_A_Rs, such as RO4938581 (Ballard et al., 2009) or the specific activation of α5GABA_A_R-targeting interneurons *in vivo*.

## Figures and Tables

**Table 1:** Time-to-peak and rise time for GABA_A_R currents (in ms).

	Time-to-peak (stimulus to peak)	Time from stimulus to 10% amplitude	Time from 10 to 90% amplitude
GABA_A,local_	17 ± 1.8	4.1 ± 0.1	7.5 ± 0.3
GABA_A,SC_	32 ± 3.1	7.8 ± 0.3	14.2 ± 0.8
GABA_A,SC_(gz)	75 ± 3.5	27.4 ± 1.1	30 ± 0.9
